# Polymorphisms in intron 1 of the *EGFR* gene in non-small cell lung cancer patients

**DOI:** 10.3892/etm.2012.681

**Published:** 2012-08-23

**Authors:** MASAYUKI SHITARA, HIDEFUMI SASAKI, KEISUKE YOKOTA, KATSUHIRO OKUDA, YU HIKOSAKA, SATORU MORIYAMA, MOTOKI YANO, TOMOYA KAWAGUCHI, AKIHITO KUBO, MINORU TAKADA, NAOTO KITAHARA, MEINOSHIN OKUMURA, AKIHIDE MATSUMURA, KEIJI IUCHI, YOSHITAKA FUJII

**Affiliations:** 1 Department of Oncology, Immunology and Surgery, Nagoya City University Graduate School of Medical Sciences, Nagoya 467-8601;; 2 Departments of Internal Medicine and; 3 Surgery, National Hospital Organization, Kinki-chuo Chest Medical Center, Sakai, Osaka 591-8555, Japan

**Keywords:** epidermal growth factor receptor, polymorphism, lung cancer, gefitinib therapy, 8227G/A

## Abstract

The epidermal growth factor receptor (*EGFR*) gene is highly polymorphic and its expression and activity may be affected by various polymorphisms. There have been several studies examining associations between *EGFR* polymorphisms and clinical outcome of lung cancer therapy; however, the underlying mechanism is largely unknown. The present study investigated *EGFR* polymorphism status and its correlation with clinicopathological features in Japanese non-small cell lung cancer (NSCLC) patients. We investigated 5 polymorphisms in the *EGFR* gene (−216G/T, −191C/A, 8227G/A, D994D and R497K) in 274 surgically-treated NSCLC patients. TaqMan single nucleotide polymorphism (SNP) genotyping assays and a PCR-based assay were used to analyze these polymorphisms. In our cohort of patients we did not find any evidence of the −191C/A polymorphism. Our results showed that the patients with the 8227GA or AA type in intron 1 had a significantly better prognosis with the anti-EGFR therapy than the patients with the GG type (p=0.0448) in terms of recurrence of lung cancer. No significant association was observed between 3 other SNPs (−216G/T, D994D and R497K) and clinicopathological features. The *EGFR* 8227G/A polymorphism in intron 1 may be associated with clinical outcome in NSCLC patients treated with EGFR tyrosine kinase inhibitors.

## Introduction

Lung cancer is a major cause of mortality from malignant diseases due to its high incidence, malignant behavior and lack of major advancements in treatment strategy (1). There is a large body of accumulated evidence that the epidermal growth factor receptor (EGFR) and its family members are heavily involved in the development and progression of numerous human tumors, including lung cancer (2,3). The EGFR tyrosine kinase inhibitor (TKI), gefitinib, was approved for the treatment of non-small cell lung cancer (NSCLC) in Japan in 2002. Two original studies have revealed that *EGFR* mutation status at the tyrosine kinase (TK) domain in NSCLC patients was correlated with a good response to gefitinib (4,5). From the results of the Iressa Pan-Asia Study (IPASS), *EGFR* mutations are the strongest predictive biomarker for progression-free survival (PFS) and tumor response to first-line gefitinib therapy for NSCLC (6).

The *EGFR* gene is highly polymorphic and its expression and activity are significantly affected by various polymorphisms (7–9). As for interethnic differences in CA repeat length in intron 1, a length of less than 17 in Japanese individuals is less frequent than in Caucasians (10). However, the frequency of *EGFR* mutations is higher in the Japanese population than in other ethnic groups. In intron 1, the −216G/T and −191C/A polymorphisms in the *EGFR* promoter are associated with altered promoter activity and gene expression (8). CA simple sequence repeats (CA-SSRs) in intron 1 (rs45559542) (8,12,13), −216G/T (rs712829) (8,12) and D994D (rs2293347) (11) polymorphisms have been reported to influence clinical outcomes in gefitinib-treated NSCLC patients. In addition, the 8227G/A polymorphism (rs763317) located in intron 1 has been reported to be associated with smoking status and gender in lung adenocarcinomas in the Taiwanese population (14).

To determine the *EGFR* polymorphism status and its correlation with clinicopathological features in lung carcinoma in the Japanese population, we investigated *EGFR* gene status using TaqMan single nucleotide polymorphism (SNP) genotyping assays. These findings were analyzed in relation to the clinicopathologic features of lung cancer.

## Materials and methods

### Patients and treatment

The study group included 261 lung cancer patients who had undergone surgery at the Nagoya City University Hospital, Japan, between 1997 and 2011. Thirty-three patients were treated with gefitinib for the recurrence of lung cancer following surgery. We also investigated polymorphisms for 13 NSCLC patients who had been treated with gefitinib for the recurrence of cancer at the Kinki-chuo Chest Medical Center, Osaka Japan. The lung tumors were classified according to the general rule for clinical and pathological recording of lung cancer in Japan, as well as according to the WHO classification. All tumor samples were immediately frozen and stored at −80°C until assayed.

The clinical and pathological characteristics of the 274 lung cancer patients were as follows: 194 (70.8%) were male and 80 were female; 192 were diagnosed as adenocarcinoma and 82 were diagnosed as other types of carcinoma (63 squamous cell carcinomas, 6 adenosquamous carcinomas, 6 large cell carcinomas, 3 carcinoids, 3 pleomorphic carcinomas, 1 adenoid cystic carcinoma and 1 carcinosarcoma); 187 (68.2%) were smokers (current or former smoker) and 87 were non-smokers (Table I). Written informed consent was obtained from the patients and the Institutional Ethics Committee of the Nagoya City University approved the study.

### Genotyping assays for the EGFR polymorphism

Genomic DNA was extracted from peripheral blood (n=109) taken prior to surgery or from adjacent normal lung tissues taken at surgery using the Wizard SV Genomic DNA Purification system (Promega Corp., Madison, WI, USA) according to the manufacturer’s instructions. *EGFR* mutation statuses at the kinase domain were investigated using the TaqMan PCR assay (Applied Biosystems, Foster City, CA, USA). The results of the TaqMan PCR assay have been previously reported (15).

TaqMan SNP genotyping assays (Applied Biosystems) were used for genotyping 4 polymorphisms in the *EGFR* gene (−216G/T, −191C/A, 8227G/A, assay ID: C_2310200_10; and D994D, assay ID: C_15970737_20; Table II) according to the manufacturer’s instructions (16). The cycling conditions for the TaqMan SNP assays were as follows: 95°C for 10 min, followed by 40 cycles of 95°C for 15 sec and 60°C for 1 min, with a 1-min extension at 25°C following the last cycle. The R521K (rs11543848, also assigned as R497K in the literature) polymorphism was examined by the PCR-RFLP method as described previously (17). Sixty-four lung cancer samples were analyzed for *EGFR* gene amplification using fluorescence *in situ* hybridization (FISH) and the results have been previously reported (18).

### Statistical analyses

Statistical analyses were carried out using the Mann-Whitney U test for unpaired samples and Wilcoxon’s signed rank test for paired samples. Linear relationships between variables were determined by means of simple linear regression. Correlation coefficients were determined by rank correlation using Spearman’s test and the χ^2^ test. The overall survival (OS) of lung cancer patients was examined using the log-rank test. All analyses were performed using the StatView software package (Abacus Concepts Inc, Berkeley, CA, USA) and differences were considered significant when p<0.05.

## Results

### EGFR gene mutation and amplification statuses

Of the 274 patients, 42 had the deletion-type *EGFR* mutations in exon 19; 35 had the missense point mutations (5 G719S, 29 L858R and 1 L861Q) in exon 18 or 21; and 4 had exon 20 insertion mutations (15,20). Sixty-four samples were studied for *EGFR* gene amplification using FISH analyses. According to the criteria by Cappuzzo *et al*, 21 were FISH-positive and 43 were FISH-negative (19).

### EGFR polymorphisms in Japanese lung cancers

In our Japanese cohort, there was no −191C/A polymorphism and we did not perform any further analyses for this polymorphism. For rs712829 (−216G/T), 255 patients were GG, 19 were GT and no TT was found. For rs2293347 (D994D), 125 patients were CC, 110 were CT and 39 were TT. For rs11543848 (R497K), 93 were AA, 135 were GA and 46 were GG. No correlation existed between these 3 SNPs (−216G/T, GG vs. GA+AA; D994D, CC+CT vs. TT; R497K, GG vs. GA+AA) and clinicopathological features of the lung cancers.

Of the 274 patients, 87 had the 8227G/A *EGFR* variant (9 AA and 78 GA). Of these, 64 were male and 23 were female, 24 were non-smokers, 59 were smokers and 4 were unknown. Adenocarcinomas were significantly more frequent in GG-type patients (139/187, 74.3%) than in the GA- or AA-type patients (53/87, 60.9%, p=0.0331). However, the polymorphism did not correlate with gender (p=0.5687), smoking (non-smokers vs. smokers, p=0.3325), or *EGFR* mutation (p=0.1539) statuses of lung cancer (Table III). *EGFR* gene amplification as identified by FISH positivity was not correlated with polymorphism statuses, including D994D (p=0.5884), −216G/T (p>0.9999), R497K (p=0.2043) and 8227G/A (p>0.9999).

### Correlation between clinical course of lung cancer patients and EGFR polymorphisms

**Figure 1:**
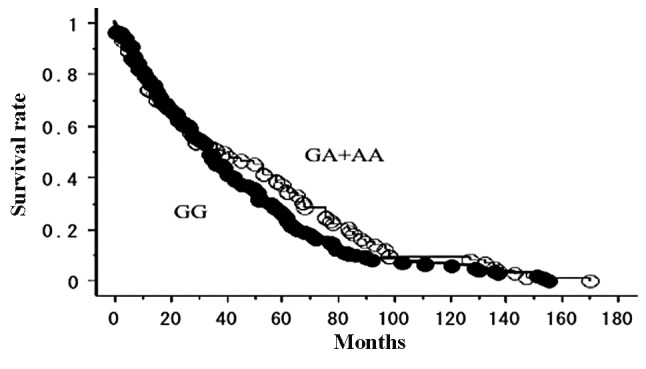
The overall survival of 225 lung cancer patients who were not treated with gefitinib was studied in reference to the *EGFR* polymorphism (8227G/A) status. The prognosis was not significantly different between the patients with 8227GG type (53/152 were deceased) and the patients with 8227GA or AA type (23/73 were deceased) (log-rank test, p=0.1753). *EGFR*, epidermal growth factor receptor.

The OS of the 225 lung cancer patients who did not receive gefitinib, with follow-up until June 30, 2011, was studied in reference to the *EGFR* polymorphism status. The prognosis was not significantly different between the *EGFR* 8227G/A types (GA+AA, 23/73 were deceased; GG, 53/152 were deceased; p=0.1753; Fig. 1). No significant association was observed between the other 3 SNPs (−216G/T, D994D and R497K) and disease outcome (data not shown).

### Correlation between clinical course of gefitinib-treated lung cancer patients and EGFR polymorphism

**Figure 2:**
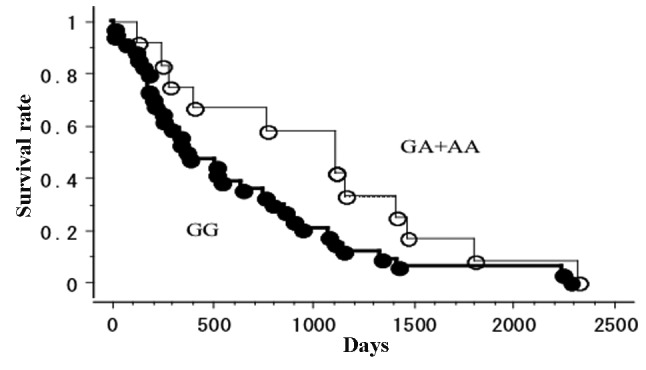
The overall survival of 46 gefitinib-treated lung cancer patients was studied in reference to the *EGFR* polymorphism (8227G/A) status. The patients with 8227GA or AA type (5/12 were deceased; median follow-up, 33.3 months) had significantly better prognosis than the patients with 8227GG type (26/34 were deceased; median follow-up, 20.0 months) (log-rank test, p=0.0448). *EGFR*, epidermal growth factor receptor.

The OS of 46 gefitinib-treated lung cancer patients, with follow-up until June 30, 2011, was studied in reference to the *EGFR* polymorphism status. In this analysis, 12 patients had *EGFR* 8227GA or AA types. Of the 46 patients, 31 had *EGFR* mutations and 11 were *EGFR* 8227GA or AA. There was a tendency towards higher *EGFR* mutation ratio in the 8227GA- or AA-type patients compared with GG-type patients (p=0.0702). Other clinical backgrounds, including gender (p=0.3071), smoking (p=0.4893) and pathological status (p=0.3059) were not correlated with 8227G/A polymorphism status. The prognosis following gefitinib therapy was significantly better for the *EGFR* GA- or AA-type patients (5/12 were deceased; mean survival, 1,014 days) when compared with the 8227GG-type patients (26/34 were deceased; mean survival, 607 days; log-rank test, p=0.0448; Fig. 2). Using the multivariate analysis, *EGFR* mutation (p=0.0316; hazard ratio, 2.174) but not 8227G/A (p=0.2232; hazard ratio, 1.587) was the independent prognostic factor for gefitinib-treated patients.

There was no association between the other 3 polymorphisms (−216G/T, p=0.7599; D994D, p=0.1813; and R497K, p=0.885) and prognosis for the gefitinib-treated patients.

## Discussion

In this study, gefitinib-treated patients with an A allele at the *EGFR* 8227G/A site were found to have a better prognosis compared with GG-type patients. However, there was no association between the other 3 polymorphisms (−216G/T, D994D and R497K) and prognosis following gefitinib therapy.

Previous studies have suggested that −216G/T (8,12) and D994D (11) polymorphisms are associated with clinical outcome of gefitinib therapy. In intron 1, CA-SSR of *EGFR* has been the most studied polymorphism. CA-SSR has been associated with *EGFR* gene expression and has been reported to correlate with clinical outcome of gefitinib therapy (8,12,13,21). Shorter CA repeats have been associated with higher transcription levels of *EGFR* and have been reported to be correlated with better clinical outcome of gefitinib therapy. Tiseo *et al* revealed that patients with the CA-16 genotype had a longer survival compared with those with other genotypes (13). Liu *et al* found that the −216G/T polymorphism and CA-19 genotype are found more frequently in patients with exon 19 deletions (22). On the other hand, Suzuki *et al* reported that the *EGFR* protein expression level was significantly higher in the shorter CA repeats group than in the longer allele group, but its length was not associated with *EGFR* somatic mutations (23). In a Japanese cohort, Ichihara *et al* reported that patients with a short CA-SSR1 had a prolonged OS as compared with those with a longer CA-SSR, but this difference was not significant in patients with a drug-sensitive *EGFR* mutation (p=0.13) (24). They found that FISH status, CA-SSR1 length and the SNP status in the promoter region (−216G/T or −191G/A) had no association with responsiveness to gefitinib in cases of lung cancer in Japanese individuals, similar to our results. One explanation for the results is that the variant forms of the SNPs, −216 G/T (6.6%) and −191G/A (0.6%), were less frequent in East Asians than in individuals of European descent (60.3 and 37%, respectively) (25). As for the D994D polymorphism, using direct sequencing, our group has previously revealed that the polymorphism did not affect the gefitinib sensitivity in Japanese individuals (26). This polymorphism is located in exon 25 and a synonymous SNP does not change the amino acid sequence of the protein, so it does not influence the biological function of the protein itself. Ma *et al* revealed that the D994D polymorphism did affect PFS but not OS following gefitinib therapy (11).

The 8227G/A polymorphism is also located in intron 1, but there have been few studies examining this SNP (14,27). Jou *et al* revealed that the *EGFR* 8227G/A polymorphism was associated with lung cancer, especially in non-smoking female lung adenocarcinoma patients in the Taiwanese population (14). Thus, this variation may lead to the different modifications of cancer genes, including *EGFR*, in tumorigenic pathways among different histological subtypes, gender and ethnicity. The 8227G/A SNP is located in intron 1, 6.9 kb downstream of the CA-SSR1 polymorphism. Additional functional analyses of this SNP are needed to better understand the mechanism by which the 8227G/A SNP of *EGFR* affects lung cancer. In our analysis, although the 8227G/A polymorphism in intron 1 was not correlated with *EGFR* somatic mutations, the GA or AA type was associated with longer survival of the gefitinib-treated patients. The underlying mechanisms remain unclear, but it may be that intron 1 of *EGFR* is associated with sensitivity to EGFR TKIs in lung cancer patients, and is correlated with certain biomarkers other than *EGFR* mutations. The sample size of the present study was too small to address this hypothesis. The extact effect of the polymorphism on survival time of patients treated with or without EGFR TKIs needs further clinical investigation with a larger sample size.

In summary, the 8227G/A polymorphism of *EGFR* may influence OS in gefitinib-treated lung cancer patients.

## Figures and Tables

**Table I:** Clinical and pathological characteristics of the 274 lung cancer patients.

	Patients (n=274)	
	No.	%
Age (years)		
≤60	78	28.5
>60	196	71.5
Gender		
Male	194	70.8
Female	80	29.2
Smoking status		
Non-smoker	87	31.8
Smoker	187	68.2
Pathological subtype		
Adeno	192	70.1
Other	82	29.9
*EGFR* mutation		
Positive	81	29.9
Negative	190	70.1

Smoker, current or former smoker; Adeno, adenocarcinoma; *EGFR*, epidermal growth factor receptor.

**Table II:** Genotyping approach for polymorphism analysis of the *EGFR* gene.

Primer sequences	−216G/T (rs712829)	−191C/A (rs712830)
VIC-MGB	AGCCTCCGCCCCC	CCTCGGCCGCGTCG
FAM-MGB	CAGCCTCCTCCCCC	CCTCGGCCGCGGCG
Forward primer	CCCGCGCGAGCTAGAC	CCCCGCACGGTGTGA
Reverse primer	GGGCGCTCACACCTG	GGCTAGCTCGGGACTCC

VIC-MGB, VIC dye-labeled TaqMan MGB probe; FAM-MGB, FAM dye-labeled TaqMan MGB probe; *EGFR*, epidermal growth factor receptor.

**Table III:** Association of the *EGFR* 8227G/A polymorphism with clinicopathological data of 274 lung cancer patients.

	GG		GA+AA		
Factors	No.	%	No.	%	p-value
Age (years)					
≤60	52	27.8	26	29.9	0.7741
>60	135	72.2	61	70.1	
Gender					
Male	130	69.5	64	73.6	0.5687
Female	57	30.5	23	26.4	
Smoking status					
Non-smoker	63	33.7	24	27.6	0.3325
Smoker	124	66.3	59	72.4	
Pathological subtype					
Adeno	139	74.3	53	60.9	0.0331
Others	48	25.7	34	39.1	
*EGFR* mutation					
Positive	61	32.6	20	23.8	0.1539
Negative	126	67.4	64	76.2	

Smoker, current smoker or former smoker; Adeno, adenocarcinoma; *EGFR*, epidermal growth factor receptor.
